# A Somatic-Type Delusional Disorder Resulted in Severe Weight Loss With a Body Mass Index of 10.2: A Case Report

**DOI:** 10.7759/cureus.56217

**Published:** 2024-03-15

**Authors:** Anas Ibn Auf, Elsaid M Bedeer, Abdullah M Almarzoogi

**Affiliations:** 1 Psychiatry, Erada and Mental Health Complex, Taif, SAU; 2 Psychiatry, Eastern Sudan College for Medical Sciences and Technology, Port Sudan, SDN; 3 Medicine, Erada and Mental Health Complex, Taif, SAU

**Keywords:** bmi, hypochondriacal psychosis, body mass index, weight loss, somatic delusion. delusional disorder

## Abstract

Weight loss is not uncommon in the field of psychiatry; however, when severe cases arise, it necessitates attention and thorough evaluation for accurate diagnosis and appropriate treatment. This report highlights the case of a 32-year-old man referred to psychiatry due to significant weight loss. The patient mentioned consuming small food portions, attributing it to an undetected stomach illness despite repeated investigations by previous treating doctors. His current weight stood at 31 kg, with a body mass index (BMI) of 10.2 kg/m². Physical examinations and laboratory investigations were otherwise within normal parameters. A somatic delusion was confirmed, and the patient has been diagnosed with a delusional disorder after excluding other possibilities. This report highlights the importance of considering delusional disorder (somatic type) as a potential diagnosis for substantial weight loss, and it records an unexpected degree of physical well-being despite a notably low BMI.

## Introduction

A delusion refers to a persistent, incorrect belief based on an inaccurate understanding of external reality, even in the face of contradictory evidence. This belief is inconsistent with one's cultural or subcultural norms, and the majority of individuals recognize it as untrue [1]. A diagnosis of delusional disorder is established when an individual experiences one or more delusional thoughts persisting for a month or longer. The diagnosis is made after ruling out physiological, substance-induced, medical, or other mental health conditions as explanations [2]. Cultural beliefs play a crucial role in this assessment, influencing both the nature of delusions and the consideration of an individual's cultural context. Apart from the presence of delusions, overall functionality remains unaffected, and observable behavior does not exhibit clear signs of oddity [2,3].

The commonly encountered types of delusional disorder include: delusional jealousy, when the patient believes that one's sexual partner is unfaithful; erotomanic type, when the patient believes that an individual, often of higher status, is in love with him or her; Grandiose type, which entails a strong conviction of possessing exceptional talent, making groundbreaking discoveries, having inflated self-worth, wielding significant power, or possessing extraordinary knowledge; the persecutory type, which includes the belief of being conspired against, attacked, harassed, or observed; and the somatic type, which involves delusions related to bodily functions and sensations [3,4].

Delusional disorder is a comparatively uncommon condition characterized by a later onset in age compared to schizophrenia, and it does not exhibit a gender predominance. Individuals with this disorder tend to be relatively stable. Despite ongoing research, the precise cause of delusional disorder remains unknown [2-5].

The somatic type of delusional disorder, historically referred to as mono-symptomatic hypochondriacal psychosis (MHP), has been a subject of study since its initial characterization by Munro in 1978 [6]. Over the years, researchers and clinicians have grappled with terminological nuances, leading to its eventual classification within the broader category of delusional disorders in the Diagnostic and Statistical Manual of Mental Disorders, DSM-IV and DSM-V [4,7]. In this type of disorder, the patient maintains a strongly fixed belief in having the symptoms. Predominantly observed among somatic delusions are themes such as delusion of infestation, body dysmorphic delusion, and delusion of body odor. These patients also have anxiety, nervousness, or other psychiatric symptoms that occur as a reaction to, or as a co-morbid disorder with, the primary psychotic illness [2,8,9].

We are introducing a case wherein the patient had a somatic type of delusional disorder, believing he had a stomach illness that necessitated restricting his food intake to prevent worsening the condition. Over time, this has led to a severe loss of weight.

## Case presentation

A 32-year-old man was referred to psychiatry after being seen by the gastroenterology department. He had complained of abdominal pain for about four years, preventing him from eating substantial amounts except for small portions of chicken meat and fat-free milk. At times, he ceased eating altogether, consuming only 'Zamzam' water, the sacred water in Islamic culture, resulting in a weight loss of more than 30 kg, with approximately 15 kg shed over the past few months.

The patient reported feeling low, fatigued, and devoid of enjoyment in life due to this ongoing illness. He denied any desire to lose weight or perceive himself as overweight. There was no reported history of induced vomiting or binge eating. He had no known psychiatric disorders and had not received psychiatric medications in the past. His medical history was unremarkable, with no diabetes, hypertension, or chronic illnesses. An upper gastrointestinal endoscopy was conducted several times. The last one was just before referral to psychiatry, revealing no significant findings related to his gastrointestinal issues.

There was no family history of mental illness. The patient was single, unemployed, and had completed education up to secondary school.

Regarding his mental status examination on admission to the psychiatry ward, the patient was irritable and potentially aggressive. His speech was coherent, with relevant answers. He exhibited a low mood with restricted affect, and there were neither formal thought disorders nor delusions, except for somatic delusions concerning an undiagnosed abdominal illness, particularly a gastric ulcer. He did not express suicidal or homicidal ideation, and there were no hallucinations from any modality. While his cognitive functions were within the normal range, he displayed impaired insight and poor judgment.

On physical examination, the patient appeared very thin and older than his age, though he did not exhibit pallor, jaundice, or cyanosis. He measured 174 cm tall and weighed 31 kg, resulting in a body mass index (BMI) of 10.2 kg/m². Other aspects of the physical examination did not reveal abnormalities, including the abdominal examination.

Routine investigations were conducted, including a complete blood picture, urine examination, psychoactive drug screening, ECG, chest X-ray, and tests for renal, liver, and thyroid function. All results were within normal limits, except for vitamin deficiency (Table 1).

**Table 1:** The results of CBC, liver function test, renal function test, serum electrolytes, serum glucose, and lipid profile. WBC: white blood cell, RBC: red blood cell, HGB: hemoglobin, HCT: hematocrit, PLT: platelet, ALP: alkaline phosphatase, ALT: alanine transaminase, BUN: blood urea nitrogen, NA: sodium, K: potassium, CL: chloride, ALB: albumin, GGT: gamma-glutamyl transferase, LDL: low-density lipoprotein, HDL: high-density lipoprotein.

Test name	Result	Conclusion	Normal range
WBC	5.84	Normal	3.5–10 × 10^3^/µL
RBC	5.05	Normal	3.5–5.5 × 10^6^/µL
HGB	14.8	Normal	12–16 q/dl
HCT	42.4	Normal	35–46%
PLT	429	Normal	150–450 × 10^3^/µL
F. random glucose	4.66	Normal	4.1–6.4 mmol/L
K	4.53	Normal	3.5–5.1 mmol/L
NA	140	Normal	136–145 mmol/L
CL	100	Normal	98–107 mmol/L
BUN	3.10	Normal	2.7–8.1 mmol/L
CREA	69	Normal	44–106 µmol/L
ALP	114	Normal	35–129 U/L
ALT	23.50	Normal	5–41 U/L
ALB	45	Normal	35–50 q/L
TP	82.90	Normal	66–87 q/L
AST	24.20	Normal	5–40 U/L
GGT	25	Normal	5–61 U/L
Total bilirubin	2.50	Normal	0.1–21 umol/L
Triglycerides	2.25	Normal	0.5–2.3 mmol/L
Cholesterol	3.85	Normal	2.12–5.2 mmol/L
LDL	2.14	Normal	0.1–2.6 mmol/L
HDL	1.06	Normal	0.9–4 mmol/L
Vit B12	99.10	Low	145–569 pmol/L
Vit D	32.89	Low	75–250 nmol/L

The patient was admitted with the initial consideration of severe depression with psychotic features, leading to the suggestion of electroconvulsive therapy (ECT) treatment. However, following a comprehensive assessment, the diagnosis was revised to delusional disorder with secondary depression. After admission, the patient was initiated on oral olanzapine at 5 mg once daily, gradually increasing to 15 mg once daily. Additionally, paroxetine was prescribed at 12.5 mg and later increased to 25 mg per day.

At the beginning of admission, the patient refused most types of food except for Zamzam water. However, after two weeks, the patient began consuming various foods, albeit in small quantities. By the third week of starting medication, notable improvements were observed in mood, affect, and the patient's attitude toward most foods. The patient's conviction regarding having a gastric ulcer decreased, and there was an observable improvement in his eating behavior compared to earlier patterns, leading to a weight gain of approximately 6 kg.

## Discussion

This case manifests an extreme state of weight loss due to somatic delusion. The patient was convinced that he had a gastric ulcer, leading to an inability to eat. This delusion persisted for about four years, resulting in an insidious but severe loss of weight with a BMI of 10.2 kg/m². The patient used to eat only small amounts of food accompanied by Zamzam water, which is sourced from the Zamzam well in Makkah, the most sacred city in Islam. In Islamic culture, Zamzam water is believed to have special properties, such as curing some illnesses and promoting health. Thus, our patient sought to address his stomach issues through this practice. 

The two main issues discussed in this case are the differential diagnosis and the notion that the patient experienced severe weight loss without manifesting serious or life-threatening physical complications.

Regarding the diagnosis of the case, a list of possible diagnoses can be considered, including anorexia nervosa, major depressive disorder, delusional disorder (somatic type), body dysmorphic disorder, somatoform disorder (somatic symptoms and related disorders), and schizophrenia. The possibility of the patient having a real abdominal illness, such as a peptic ulcer, was excluded based on the assessment and investigation by the gastroenterology team. An upper GI endoscopy revealed no evidence of ulcers.

Anorexia nervosa was ruled out as the patient does not exhibit an intense fear of gaining weight or becoming fat, and there is no body image disturbance. The delusional nature of the patient’s thoughts excluded the diagnoses of body dysmorphic disorder and somatoform disorder (somatic symptoms and related disorders). Schizophrenia was also excluded since the diagnostic criteria were not met; there was only one 'criterion A' symptom according to DSM-5 [4].

Severe depressive disorder with psychotic manifestations was considered a provisional diagnosis based on the presence of depressive symptoms along with somatic delusions. However, it became evident that the somatic delusion preceded the appearance of mood symptoms. The final diagnosis of delusional disorder (somatic type) was made, as it requires at least one month of persistent somatic delusions with no other psychotic symptoms after excluding other possibilities, according to DSM-5 [4]. The depressive symptoms were secondary to the primary psychotic disorder.

The other part of the discussion will be on extreme weight loss. The patient presented with a BMI of 10.2 kg/m², indicating severe underweight. This classification begins when the BMI falls below 16.5 kg/m² [10]. Underweight is linked to insufficient micronutrients, compromised immune function, osteoporosis, and asthma. Recent research indicates that being underweight is also a factor that increases the risk of cardiovascular disease [11]. It is also associated with increased susceptibility to infection and increased mortality [12,13]. Although severe weight loss often leads to physical complications, similar to what is observed in individuals with anorexia nervosa, our patient relatively maintained physical well-being. This is likely attributed to the gradual onset of the weight loss. Over the past four years, he has begun reducing his oral intake and avoiding certain foods he perceives as harmful. Throughout this period, he consistently consumed nutritious items such as chicken and milk, contributing significantly to the preservation of his overall health.

Complex metabolic adaptations may contribute to the survival of individuals with an extremely low BMI [14]. However, it remains unclear whether these metabolic adaptations result from a decrease in fat-free body mass or the activation of energy-conserving mechanisms. It is reported in some patients with a mean BMI of 15.6 kg/m² that there was a noticeable reduction in the basal metabolic rate (BMR) adjusted to fat-free mass (FFM) when compared to controls with normal body weight. Various mechanisms, such as changes in hormone levels, thyroid metabolism, insulin secretion, and leptin levels, could be accountable for alterations in energy expenditure and metabolic adaptations during weight loss [15]. Although a BMI below 13 kg/m² has been considered a poor prognosis, there are documented cases of individuals who survived with significantly lower BMIs, such as 7.8, 7.8, and even as low as 6.7 kg/m² [14,15].

## Conclusions

This report presented a case with an initially ambiguous diagnosis and explored various possible diagnoses. It emphasized the significance of contemplating delusional disorder (somatic type) as a possible diagnosis in instances of substantial weight loss. Furthermore, it documented an unforeseen level of physical health despite a very low BMI.
